# Inflammatory Bowel Disease in the Post-STRIDE II Era: Epidemiology and Long-Term Clinical Outcomes from a Population-Based Study

**DOI:** 10.3390/medsci13020055

**Published:** 2025-05-03

**Authors:** Fabio Ingravalle, Marco Valvano, Andrea Barbara, Dorian Bardhi, Giovanni Latella, Angelo Viscido, Mariachiara Campanale, Antonio Vinci, Carlo Viora, Giampiera Bulfone, Rocco Mazzotta, Massimo Maurici

**Affiliations:** 1 Doctoral School of Nursing Sciences and Public Health, University of Rome “Tor Vergata”, 00133 Rome, Italy; 2 Local Health Authority “Roma 6”, 00041 Albano Laziale, Italy; 3 Division of Gastroenterology, Galliera Hospital, 16128 Genoa, Italy; 4 Department of Life, Health, and Environmental Sciences, Division of Gastroenterology, Hepatology, and Nutrition, University of L’Aquila, 67100 L’Aquila, Italy; 5 Local Health Authority “Roma 1”, 00193 Rome, Italy; 6 Health Management Unit, Azienda Ospedaliero Universitaria delle Marche, 60126 Ancona, Italy; 7 Azienda Regionale Emergenza Sanitaria ARES 118, 00149 Rome, Italy; 8 Department of Medical, Surgical Science, and Advanced Technology “GF Ingrassia”, University of Catania, 95123 Catania, Italy; 9 Department of Biomedicine and Prevention, University of Rome “Tor Vergata”, 00133 Rome, Italy

**Keywords:** inflammatory bowel disease, Crohn’s disease, ulcerative colitis, surgery for inflammatory bowel disease, inflammatory bowel disease epidemiology, population study

## Abstract

**Background/Objectives:** Inflammatory bowel disease (IBD) includes Crohn’s disease (CD) and ulcerative colitis (UC). The availability of an increasing number of new molecules approved for IBD treatment has increased our ability and aspirations to change the trajectory of the disease. The Selecting Therapeutic Targets in Inflammatory Bowel Disease (STRIDE) II consensus (2018) is the current suggested strategy for IBD management, which recommends a treat-to-target approach. The primary objective of this study is to describe the clinical history of IBD in the post-STRIDE II era and to quantify the burden of IBD in terms of hospitalisation rate. The secondary objective is to estimate the 6-year risk of intestinal resection among IBD patients. **Methods:** A population-based time series analysis was conducted on administrative data; retrospective data from January 2011 to December 2021 were collected for the Local Health Authority “Roma 1” population (∼1.5 million residents). Hospitalisation and surgical events were prospectively recorded for patients newly diagnosed between January 2018 and February 2022 (n = 556), with follow-up throughout May 2024. A Kaplan–Mayer survivor analysis was performed to estimate the cumulative surgery risk. **Results:** In 2021, the IBD prevalence was 218.3 cases/100,000 people (77.2 CD, 141.1 UC). The incidence trend slowly increased during the last decade, up to 5.3 (CD) and 9.4 (UC) cases/100,000 ppl/year. The yearly hospitalisation rate remained stable, near 16.5%. The 6-year cumulative risk of surgery was 36% for CD and 20% for UC. **Conclusions:** The incidence of IBD has increased in the last few decades, with substantial stability in regard to the incidence of surgery and hospitalisations. Thus, the current IBD management approach has only had a small effect on changing the natural history of the disease.

## 1. Introduction

Inflammatory bowel diseases (IBDs), including Crohn’s disease (CD) and ulcerative colitis (UC), are chronic inflammatory conditions with a multifactorial etiopathogenesis affecting the gastrointestinal system and are characterised by periods of relapse and remission and a progressive nature, leading to bowel damage and disability [1,2]. Clinical, endoscopic, and radiologic assessments are often used to predict long- and mid-term patient outcomes [3,4].

The burden of IBD is increasing worldwide and, in the past decade, IBD has emerged as a public health challenge [5,6]. The estimated prevalence (>0.3%) of IBD continues to rise in Western countries, with a high burden of disease in North America, Oceania, and Europe, while newly industrialised countries in Asia, Africa, and South America are showing a progression in terms of IBD prevalence, as they become more westernised and urbanised [7].

Four epidemiological stages of IBD have been proposed to explain the evolution of IBD across the epidemiologic transition periods. The four stages are as follows: (1) emergence, (2) acceleration of incidence, (3) compounding prevalence, and (4) prevalence equilibrium. Western countries (i.e., most countries in North America, Western Europe, and Oceania) are in stage 3 (compounding prevalence), where IBD prevalence exceeds 0.5%. Newly industrialised countries in Asia, Latin America, and the Middle East are in stage 2 (acceleration of incidence), with a low prevalence but rapidly increasing incidence [8].

In the last decade, a paradigm shift in IBD management has occurred [9]. Both quality of life (QoL) and clinical endpoints are now a target of interventions. Prevention measures (such as smoking prevention, reducing stress factors, and a particular focus on younger age groups) have been proposed in recent times that have a debatable role on the causal effects of the disease, but that have had a notable impact on symptom reduction [10,11,12]. Alternative measures for QoL improvements have also been proposed, such as telemedicine, the use of which is already suggested for patient monitoring and supervision by several clinical studies, and is even recommended in a recent position paper by the European Federation of Internal Medicine [13,14]. On the pharmacological front, the availability of an increasing number of new molecules (including adjuvants, biological therapies, and small molecules) has increased our ability and aspirations to reach beyond the conventional treatment goals, such as clinical remission [15,16,17]. Despite the current therapies available, a proportion of patients experience relapses and continuous inflammation, which occasionally requires the surgical removal of parts of their intestine [18]. The treat-to-target approach, designed to obtain an even more ambitious objective, represents the current strategy in IBD management [19,20]. STRIDE I, proposed in 2015, was the first consensus oriented towards this paradigmatic shift [21]. The valuable lessons gained from this consensus were subsequently developed into the Selecting Therapeutic Targets in Inflammatory Bowel Disease (STRIDE) II consensus in 2018 [22]. However, despite the novel treatments available and the new tight control strategies, including targeted and customised therapies, and the attention being paid towards the patients’ immunocompetence or immunosuppression via dedicated vaccination schedules, the burden of disease, and, in particular, the need to reduce the risk of surgery and hospitalisation, represents a significant unmet need [23,24].

### 1.1. IBD Within the Healthcare System in Italy

In regard to the Italian National Healthcare System (NHS), individuals are required to pay a contribution fee for certain health services. They do not pay the entire cost of the service; instead, they contribute a portion of the cost, and the rest is covered by the NHS. Depending on the clinical and/or socio-economic conditions, specific individuals have an exemption right in regard to the requirement to pay a contribution fee. To obtain an exemption, specific criteria must be met. In particular, for IBD patients, after an endoscopic and histological evaluation, a gastroenterology specialist can certify the illness condition by issuing a healthcare certificate that exempts the IBD patient from being required to pay the contribution fee. After a diagnosis, healthcare approval by the NHS is quite fast, usually only requiring up to two weeks [25]. After approval, all IBD-related health services are registered by the NHS as provided to the specific patient. A table of procedures that the NHS classifies as IBD-related is available in Table A1.

Concerning hospital services, Italian NHS is a Beveridge Health System which has adopted DRG payments to hospitals; Italian DRGs follow the US (CMS) model ver. 24.0. In order to obtain a DRG payment, each structure must generate and transmit a Hospital Discharge Forms (SDO—Scheda Dimissione Ospedaliera), in anonymous form, for every admitted patient [26]. SDOs are transmitted monthly to Regional Governments, who proceed to refund the hospital structures, and are also transmitted periodically to the Ministry of Health for statistical and administrative purposes.

### 1.2. Study Objectives

The primary objective of this study is to define the incidence and prevalence of IBD from 2011 to 2021. The secondary objective is to quantify the risk of surgery among IBD patients up until 2024 in order to understand whether the diffusion and application of the STRIDE II guidelines resulted in a change in IBD-related hospitalisation rates and surgery events.

## 2. Materials and Methods

### 2.1. Study Design and Reporting

This study was designed in two components. First, there is an epidemiological description of IBD in the interested territory, conducted as a population-based interrupted time series, with data collected from routinely used administrative records referring to the years 2011–2021. Second, the data of all patients with diagnosed IBD from January 2018 to February 2022 were collected and analysed prospectively. The REporting of studies Conducted using Observational Routinely-collected Data (RECORD) guidelines were used for reporting [27].

### 2.2. Population and Data Sources

The population comprises all living residents in the administrative jurisdiction of “Roma 1” Local Health Authority (LHA) in Rome, Italy, which encompasses the Northwestern sector of the urban territory of Rome, Italy. It hosts more than 15 hospital structures within its territory, including 2 directly managed hospital facilities, 3 publicly run Hospitals, and 13 Emergency Departments (out of 22 in the Rome metropolitan area) [28].

Complete data regarding new IBD diagnoses were available from January 2011 to December 2021. These data were used for incidence and prevalence estimation. Data regarding older diagnoses was available, but with no extra information regarding the diagnosis date; this was utilised only for prevalence estimation. Data regarding hospitalisation from either public or private facilities, both inside or outside the LHA Roma 1 territory, was available from January 2018 to May 2024 and was used to describe hospitalisation trends.

Data were collected in pseudo-anonymized form via the digital Business Intelligence (BI) platform routinely used in LHA Roma 1: the ID code of each person is represented by an encrypted string, yet it is still possible to connect health events attributable to the same individual. Using this pseudo-anonymized ID, information from several sources (Exemptions registry, SDO database).

This study included person-level linkage between the exemptions registry and SDO repository. Linkage was executed automatically using Roma 1 BI software. A detailed list of the administrative datasets used in this study is provided in Table A2.

**Figure 1:**
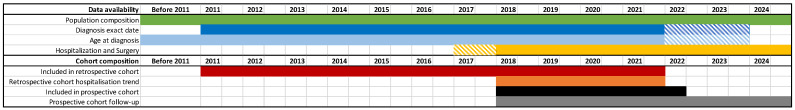
Gantt chart of data availability and cohort composition timelines. Shaded areas indicate incomplete or partial data coverage for the year.

Data availability and cohort composition are depicted in Figure 1.

### 2.3. Outcome Definitions

The date each patient obtained IBD ticket exemption was used as proxy for IBD diagnosis (there may be a few days in delay between clinical diagnosis and administrative exemption recording). Patients have a strong incentive to obtain their exemption recorded since they incur no charges for visits and drugs related to their pathology. For this reason, there is very low chance of missing diagnosis data.

For the retrospective section of the study, as per current practice in population studies, we defined an IBD-related hospitalisation based on the presence of an International Classification of Diseases, Ninth Revision, Clinical Modification (ICD-9-CM) discharge diagnosis of either CD or UC as the most responsible comorbidity, or primary inter-service or inter-hospital transfer diagnosis [29,30].

For the prospective section of the study, surgical treatments unrelated to IBD, including infectious, biliopancreatic, and traumatic, have not been considered as IBD-related and were therefore excluded from the analysis. A comprehensive list of codes and procedures included and excluded is available in Table A3.

### 2.4. Statistical Methods

#### 2.4.1. Retrospective Analysis

The monthly incidence of IBD (distinguished as either UC or CD) among the general population in LHA Roma 1 was calculated for each age range (stratified in 10-year age groups) and for each observation year from January 2011 to December 2021; standardisation was performed based on the 2024 LHA Roma 1 population composition. Age-standardised incidence was calculated annually, and separate linear regression models were used to test for any statistically significant increases observed during the 10-year timespan. As a sensitivity analysis, monthly data were filtered using the Baxter–King method for time series decomposition [31]. The trend component was isolated and compared with actual observations (after the flat mean of residuals was added back, to correct for underestimation due to filtering out both stochastic and cyclic components) following simple smoothing using a 3-period simple autoregressive moving average (3-ARMA).

The monthly hospitalisation trend among the cohort of IBD patients residing in Roma 1 (N = 2191) was calculated from January 2018 to May 2024 using time series decomposition, as per primary analysis. The hospitalisation trend was also described as a yearly percentage among IBD patients.

Statistical analysis was conducted at the Department of Biomedicine and Prevention, University of Rome “Tor Vergata”. MS Excel v.2016 and Stata v.17 were used for calculations and graph creation.

#### 2.4.2. Prospective Analysis

A cohort of 556 patients diagnosed from January 2018 to February 2022 was included in the prospective analysis. All hospitalisations were screened until May 2024, and recorded interventions were considered as per primary outcome if a relevant ICD-9-CM procedure was registered. A table of relevant procedures is available in Table A3.

The Kaplan–Meier function was then proposed to estimate the cumulative risk for surgery among IBD patients, both in raw data and after adjustment for gender and age at diagnosis via a Cox proportional hazards model.

#### 2.4.3. Bias and Bias Reduction

A possible source of bias lies in the underestimation of actual IBD cases due to misdiagnosis or difficult diagnosis, especially for people coming from rural areas, where IBD may require differential diagnosis with other gastrointestinal conditions. The risk of our results being significantly affected is, however, very low in magnitude because most of the included population comes from a heavily urbanised area and because difficult-to-diagnose IBD should contribute minimally to overall hospitalisations and treatment effects.

Another possible source of bias is linked to the retrospective nature of this study, as we could not select patients by excluding confounding factors. No information was available regarding clinical presentation or specific risk factors such as drinking or smoking habits; however, adjustment for gender and age at diagnosis was possible, and, as such, we performed it in the inferential analysis.

## 3. Results

### 3.1. IBD Epidemiology

**Figure 2:**
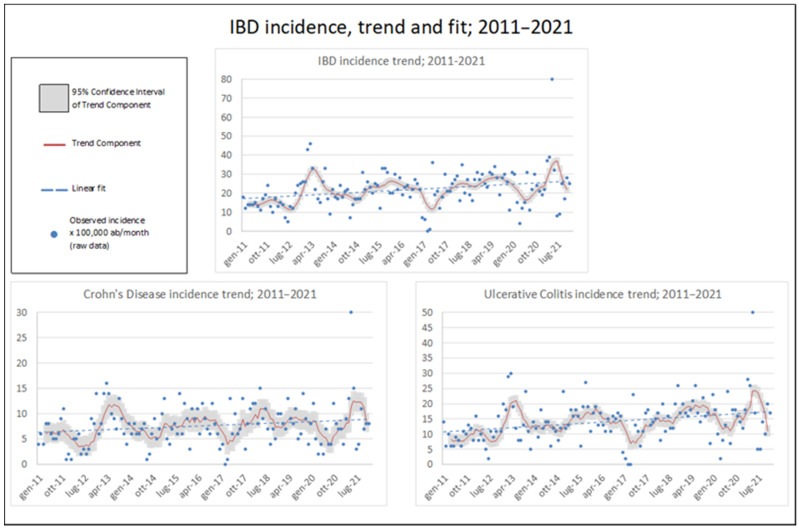
IBD monthly incidence (×100,000 ppl) with trend component (with 95% Confidence Interval) and linear fit; 2011–2021.

**Figure 3:**
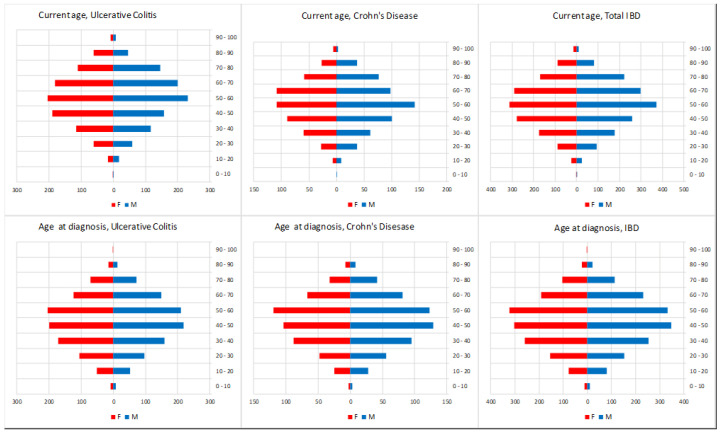
IBD prevalence at May 2024: age and gender composition.

**Figure 4:**
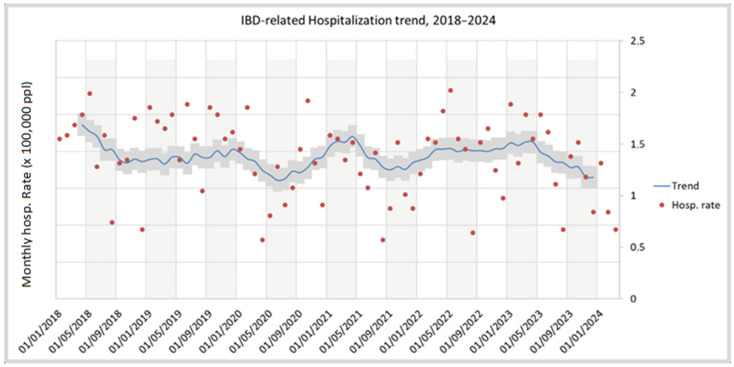
Unadjusted IBD-related hospitalisations, 2018–2024.

Details on yearly IBD incidence are provided in Table 1. A total of 2859 patients were diagnosed with IBD from January 2011 to December 2021. The raw incidence of IBD increased slowly in the observed period: new diagnoses of IBD rose from 184 in 2011 to 341 in 2021, increasing from 14.7 to 28.5 × 100,000 ab per year (average: 21.2). Figure 2 depicts monthly observations and trend components, filtered for cyclical effects via the Baxter–King method. Figure 3 illustrates the population composition by age and gender, both currently and at the time of the diagnosis. For the 2191 patients diagnosed since 2011 and currently residing in Roma 1, the hospitalisation trend in the last 6 years was calculated (Figure 4). The raw hospitalisation rate remained relatively constant throughout the study period, with 1.38 hospitalisations/100,000 ppl per month. This roughly translates to 16.5% yearly hospitalisation rate among IBD patients. The prevalence of IBD is estimated to be 218.3 cases/100,000 ppl, with 77.2 cases of CD and 141.1 cases of UC.

### 3.2. Prospective Cohort

Of the 556 patients constituting the prospective cohort, 194 (34.9%) had CD and 362 (65.1%) UC. Demographic details are available in Table 2. The mean follow-up time was approximately 6 years (SD: 10 months).

Among the 196 CD patients (110 M, 86 F), 19 (9.8%) surgery events occurred. In total, 2 of them coincided with the diagnosis moment; thus, acute presentation can be estimated to happen in 1.0% of all cases. The Kaplan–Meier function shows a 27% cumulative risk of surgery at 6 years from diagnosis, after adjusting for gender and age at diagnosis, while unadjusted estimates indicate a 12% cumulative risk of surgery after 6 years.

**Figure 5:**
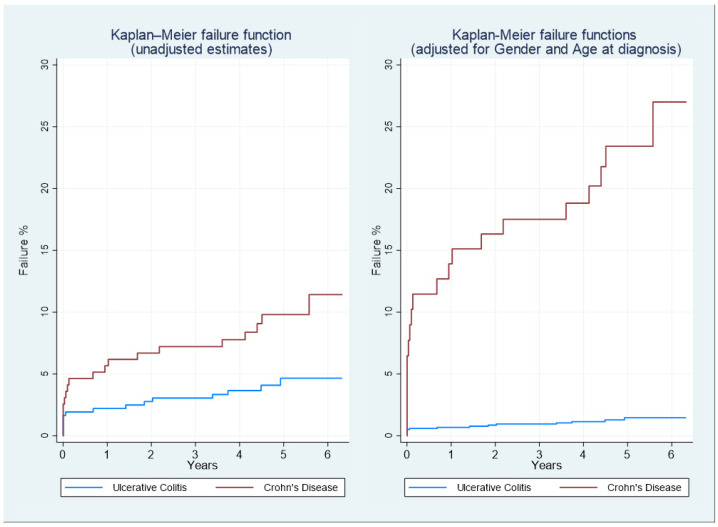
Kaplan–Meier failure functions for Ulcerative Colitis (UC) and Crohn’s Disease (CD) patients, raw data (**left**), and adjusted for age at diagnosis and gender (**right**). The outcome of interest is the first occurrence of surgery.

Among the 362 UC patients (190 M, 172 F), 15 (4.1%) surgery interventions occurred, with acute presentation observed in 12 cases (3.3% of patients). Adjusted Kaplan–Meier function shows a 2% cumulative risk of having surgery at 6 years, while observed surgery cumulative risk was close to 5% (Figure 5).

## 4. Discussion

### 4.1. Key Results

A small increase in IBD incidence in the last decade was observed in our population study, with an average yearly estimated incidence of 21.2 cases ×100,000 ppl. This increasing trend has also been noted elsewhere, for both CD and UC [6,32,33]. The reasons for this increase are largely unknown. Both improved diagnostic accuracy and greater patient awareness may have contributed to the rise in new IBD diagnoses. Nevertheless, unknown environmental factors might also play a key role in increasing IBD prevalence [34].

Robust data from a review of population-based studies (in the pre-STRIDE era) demonstrate that between 27% and 50% of CD patients require surgery between 5 and 10 years after diagnosis, respectively, with a hospitalisation rate of 20% per year. However, in these studies, no data from the biological era were available [35,36]. In our study, the Kaplan–Meier analysis showed that the cumulative 6-year risk of surgery was 27% and 2% from diagnosis in CD and UC, respectively (adjusted for gender and age at diagnosis), while the hospitalisation rate was 16.5% for IBD patients.

### 4.2. Strengths and Limitations

A significant strength of this study is the use of data with little to no missing records. This was possible because informatics systems do not allow for the treating physician to proceed with a medical record when information on exemption, diagnosis, or clinical procedure is missing; also, the physician is warned when apparent incongruences are detected by the system (such as a surgical procedure registered within a psychiatric ward DRG), allowing them to correct occasional mistakes. Moreover, use of population databanks allowed us to have a minimal risk of excluding actual IBD patients from our analysis. This is also true for procedures where the patients were exempt from paying a contribution fee, as the prescription charge exemption is recorded and linked to the specific procedure.

Another strength of this study is that, during the considered period, no substantial modifications in IBD management strategy occurred. No new molecules were approved, and the STRIDE II approach was proposed at the beginning of the enrolment period. This aspect makes the reported results extremely representative of the current situation. However, baseline characteristics concerning relevant factors that can contribute to the risk of surgery, such as smoking habits and disease patterns, were not available in our database.

### 4.3. Interpretation and Generalisability

The availability of an increasing number of new molecules approved for IBD treatment has heightened our aspirations to alter the disease’s natural history. Changes in the paradigm of IBD management with treat-to-target strategies and the spread of the STRIDE approach are significant sources of bias that make it difficult to evaluate the role of biological therapies in the decline of IBD-related surgery and hospitalisation. Likewise, the widespread use of new therapies affects the changing incidence of these outcomes.

In recent times, few population-based studies have attempted to address the evolution of IBD-related surgery and hospitalisation over the years. A UK population-based cohort study evaluated the 5-year risk of surgery in four cohorts followed from 2000 to 2017. The 5-year cumulative risk of surgery was 20.4% in cohort 1 (2000–2004), 18.3% in cohort 2 (2005–2008), 14.7% in cohort 3 (2009–2013), and 13.0% in cohort 4 (2014–2017). Conversely, the prevalence of biological therapies prescriptions increased in each of the cohorts considered. Thus, the authors concluded that increased and earlier use of biologic therapy in CD patients corresponded with a decreasing requirement for surgery over time [37]. On the other hand, a Canadian population-based interrupted time series study evaluated the effect of the marketplace introduction of infliximab on population rates of hospitalisations and surgeries. Infliximab did not produce significant declines in the rates of CD-related hospitalisations (OR 1.06, 95% CI from 0.811 to 1.39) or surgeries (OR 1.10, 95% CI from 0.810 to 1.50), or in the rates of UC-related hospitalisations (OR 1.22, 95% CI from 1.07 to 1.39) or colectomies (OR 0.933, 95% CI from 0.54 to 1.61) [29]. In Italy, another retrospective study conducted over a different population showed that the introduction of biological therapies had only a slight impact on the occurrence of surgery in CD patients over a long observation period, contributing to delaying the first intestinal resection [38]. Furthermore, despite the increased post-operative use of anti-TNFα agents in CD patients in the last two decades, the impact of this strategy on the risk of the long-term re-operation rate has been modest [39].

## 5. Conclusions

A slight increase in the raw incidence of IBD has been observed in recent decades, with substantial stability in the incidence of surgeries and hospitalisations. However, the estimated incidence and prevalence of IBD are still slightly lower than in other Western countries. This could be due to underdiagnoses or an actual lower incidence among the Italian population. Hospitalisation and surgery rates have also remained stable over the past decade. Therefore, changes in current IBD management appear to have had only a small effect on altering the natural history of the disease.

## Figures and Tables

**Table 1:** Raw incidence of IBD by age range and standardised incidence × 100,000 ab (ref. population: 2024 LHA Roma 1 residents); 2011–2021. UC: Ulcerative colitis. CD: Crohn’s Disease.

IBD Cases	Age at Diagnosis											Age-Standardised Incidence × 100,000 PPL		
Year	0–10	10–20	20–30	30–40	40–50	50–60	60–70	70–80	80–90	90–100	Total	UC	CD	IBD
**2011**	5	11	17	38	45	35	19	10	4	0	184	8.83	5.83	14.67
**2012**	2	11	15	41	39	33	24	8	2	0	175	9.22	4.81	14.03
**2013**	1	9	18	65	74	67	55	19	5	0	313	16.13	9.45	25.58
**2014**	0	12	15	36	58	53	29	10	1	0	214	11.30	5.81	17.11
**2015**	2	8	29	38	72	74	43	26	8	0	300	16.05	8.18	24.23
**2016**	0	14	28	37	48	71	54	21	7	0	280	14.63	8.19	22.82
**2017**	1	11	20	35	30	43	34	15	3	0	192	9.60	6.07	15.68
**2018**	2	16	30	50	66	71	34	27	3	0	299	15.59	9.21	24.79
**2019**	1	19	42	32	67	63	53	34	4	0	315	16.95	8.36	25.31
**2020**	2	16	35	51	46	52	25	15	4	0	246	13.99	6.16	20.15
**2021**	4	22	44	69	74	70	34	20	3	1	341	18.55	9.98	28.54
Total	**20**	**149**	**293**	**492**	**619**	**632**	**404**	**205**	**44**	**1**	**2859**	**-**	**-**	**-**

**Table 2:** Demographic composition of prospective cohort. UC: Ulcerative colitis; CD: Crohn’s Disease; IQR: Interquartile range; SD: Standard Deviation.

	CD: N = 194	UC: N = 362
Age at diagnosis (mean; SD)	47.0; 15.9	48.5; 15.8
Male (N; %)	109; 56.2%	190; 52.5%
Total interventions (N; %)	21; 10.8%	36; 9.9%
Median follow-up duration (months; IQR)	59.7 (50.9–69.0)	58.1 (49.2–65.5)

## Data Availability

Data are the property of ASL Roma 1 Public Health Authority. Data are available from the Roma 1 Institutional Data Access (e-mail at: protocollo@pec.aslroma1.it) for researchers who meet the criteria for access to confidential data.

## Abbreviations

The following abbreviations are used in this manuscript:

ARMA	Auto-Regressive Moving Average
CD	Crohn’s Disease
DRG	Diagnosis-Related Group
IBD	Inflammatory Bowel Disease
ICD-9-CM	International Classification of Diseases, 9th Revision, Clinical Modification
LHA	Local Health Authority
NHS	National Healthcare System
PPL	People
QOL	Quality of Life
SDO	Scheda di Dimissione Ospedaliera (hospital discharge form)
UC	Ulcerative Colitis

## Appendix A

**Table A1:** List of possible services provided by the NHS for free to patients with IBD diagnosis.

Health Service with Ticket Exemption	Type	Note
**CHECK-UP VISIT NECESSARY TO MONITOR THE DISEASE, THE MOST FREQUENT COMPLICATIONS AND TO PREVENT FURTHER WORSENING**		
**VENOUS BLOOD SAMPLING**	ALANINE AMINOTRANSFERASE (ALT) (GPT)	
	ALPHA 1 ACID GLYCOPROTEIN	
	ASPARTATE AMINOTRANSFERASE (AST) (GOT)	
	REFLEX BILIRUBIN (cut-off > 1 mg/dL unless more restrictive cut-offs are defined at a regional level. Included: Direct and Indirect Bilirubin	
	COBALAMIN (VIT. B12)	
	FERRITIN	
	IRON	
	FOLATE	
	ALKALINE PHOSPHATASE	
	GAMMA-GLUTAMILTRANSFERASE (gamma GT)	
	PANCREATIC LIPASE	
	POTASSIUM	
	BLOOD PROTEINS (ELECTROPHORESIS) Included: Total protein determination 90.38.5	
	SODIUM	
	TRANSFERRIN	
	BLOOD CHROME: BLOOD CYTOMETRY EXAM AND DIFFERENTIAL LEUKOCYTE COUNT Hb, GR, GB, HCT, PLT, IND. DERIV. Including any microscopic control	
	C-REACTIVE PROTEIN (Quantitative)	
	BLOOD SEDIMENTATION RATE (ESR)	
**RADIOLOGY**		
**TRADITIONAL**	DOUBLE CONTRAST COLON ENEMA	
	DOUBLE CONTRAST SMALL BOW ENEMA	
**ULTRASOUND**	ULTRASOUND OF THE COMPLETE ABDOMEN	Possible Colour-Doppler integration
	ULTRASOUND OF THE INTESTINAL LOOP	
**DENSITOMETRY**	BONE DENSITOMETRY. LUMBAR DXA	No more than 1 in 12–18 months
	BONE DENSITOMETRY. FEMORAL DXA	No more than 1 in 12–18 months
	BONE DENSITOMETRY. ULTRADISTAL DXA	No more than 1 in 12–18 months
**ENDOSCOPY**		
**UPPER SITES**	ESOPHAGOGASTRODUODENOSCOPY [EGDS]	
	BIOPSY DURING EGDS	Brushing or washing for sample collection
	BIOPSY OF THE SMALL INTESTINE DURING ENTEROSCOPY	Brushing or washing for sample collection
**LOWER SITES**	TOTAL COLONOSCOPY WITH FLEXIBLE ENDOSCOPE	
	RECTOSIGMOIDOSCOPY WITH FLEXIBLE ENDOSCOPE	
	PROCTORECTORECTOSIGMOIDOSCOPY WITH RIGID ENDOSCOPE	
	SINGLE SITE BIOPSY OF THE LARGE INTESTINE DURING TOTAL COLONOSCOPY WITH FLEXIBLE TUBE	Brushing or washing for sample collection
	BIOPSY DURING PROCTORECTOSIGMOIDOSCOPY	Brushing or washing for sample collection

**Table A2:** Roma 1 administrative databases used to capture study information.

Variable	Database
**IBD PATIENT STATUS**	Roma 1 ticket exemptions database
**IBD PATIENTS DIAGNOSIS DATE**	Roma 1 ticket exemptions database
**DEMOGRAPHIC VARIABLES**	Roma 1 healthcare-registered persons database
**SURGERY INTERVENTIONS**	Roma 1 hospital records administrative database
**HOSPITALISATIONS**	Roma 1 hospital records administrative database

**Table A3:** Table of ICD-9CM codes used as surgical intervention outcome in prospective cohort analysis. *: excluded for Ulcerative Colitis cohort.

ICD Code	Meaning
ibd-Related (included):	
* 42.33	Endoscopic Excision Or Destruction Of Lesion Or Tissue Of Esophagus
* 42.52	Intrathoracic Esophagogastrostomy
* 43.91	Total Gastrectomy With Intestinal Interposition
* 45.30	Endoscopic Excision Or Destruction Of Lesion Of Duodenum
* 45.62	Other Partial Resection Of Small Intestine
* 45.72	Open And Other Cecectomy
45.73	Open And Other Right Hemicolectomy
45.75	Open And Other Left Hemicolectomy
45.79	Other And Unspecified Partial Excision Of Large Intestine
45.8	Total Intra-Abdominal Colectomy
45.91	Small-To-Small Intestinal Anastomosis
45.93	Other Small-To-Large Intestinal Anastomosis
45.94	Large-To-Large Intestinal Anastomosis
46.02	Resection Of Exteriorized Segment Of Small Intestine
46.81	Intra-Abdominal Manipulation Of Small Intestine
48.63	Other Anterior Resection Of Rectum
48.69	Other Resection Of Rectum
49.93	Other Incision Of Anus
54.21	Laparoscopy
* 54.3	Excision Or Destruction Of Lesion Or Tissue Of Abdominal Wall Or Umbilicus
* 54.63	Other Suture Of Abdominal Wall
* 83.39	Excision Of Lesion Of Other Soft Tissue
Not IBD-Related (Excluded):	
45.16	Esophagogastroduodenoscopy [EGD] with closed biopsy
45.19	Other diagnostic procedures on small intestine
45.23	Colonoscopy
45.24	Flexible sigmoidoscopy
45.25	Closed [endoscopic] biopsy of large intestine
45.26	Open biopsy of large intestine
45.27	Intestinal biopsy, site unspecified
45.28	Other diagnostic procedures on large intestine
45.29	Other diagnostic procedures on intestine, site unspecified
48.23	Rigid proctosigmoidoscopy
48.24	Closed [endoscopic] biopsy of rectum
48.25	Open biopsy of rectum
48.29	Other diagnostic procedures on rectum, rectosigmoid and perirectal tissue
48.36	[Endoscopic] polypectomy of rectum
49.21	Anoscopy
49.29	Other diagnostic procedures on anus and perianal tissue
50.11	Closed (percutaneous) [needle] biopsy of liver
51.1	Endoscopic retrograde cholangiopancreatography [ERCP]
51.11	Endoscopic retrograde cholangiography [ERC]
54.24	Closed [percutaneous] [needle] biopsy of intra-abdominal mass
46.1	Colostomy, not otherwise specified
46.11	Temporary colostomy
46.2	Ileostomy, not otherwise specified
46.21	Temporary ileostomy
46.23	Other permanent ileostomy
46.51	Closure of stoma of small intestine
48.73	Closure of other rectal fistula
49.51	Left lateral anal sphincterotomy
49.59	Other anal sphincterotomy
0.34	Imageless computer assisted surgery
25.1	Excision or destruction of lesion or tissue of tongue
25.2	Partial glossectomy
27.29	Other diagnostic procedures on oral cavity
27.42	Wide excision of lesion of lip
27.49	Other excision of mouth
27.56	Other skin graft to lip and mouth
27.79	Other operations on uvula
30.09	Other excision or destruction of lesion or tissue of larynx
30.22	Vocal cordectomy
40.41	Radical neck dissection, unilateral
41.5	Total splenectomy
42.33	Endoscopic excision or destruction of lesion or tissue of esophagus
42.52	Intrathoracic esophagogastrostomy
43.89	Other partial gastrectomy
43.91	Total gastrectomy with intestinal interposition
44.19	Other diagnostic procedures on stomach
44.43	Endoscopic control of gastric or duodenal bleeding
44.67	Laparoscopic procedures for creation of esophagogastric sphincteric competence
44.68	Laparoscopic gastroplasty
45.13	Other endoscopy of small intestine
45.42	Endoscopic polypectomy of large intestine
46.81	Intra-abdominal manipulation of small intestine
46.85	Dilation of intestine
48.76	Other proctopexy
48.93	Repair of perirectal fistula
49.11	Anal fistulotomy
49.12	Anal fistulectomy
49.73	Closure of anal fistula
49.93	Other incision of anus
50.22	Partial hepatectomy
50.3	Lobectomy of liver
51.04	Other cholecystotomy
51.22	Cholecystectomy
51.23	Laparoscopic cholecystectomy
51.83	Pancreatic sphincteroplasty
51.98	Other percutaneous procedures on biliary tract
54.11	Exploratory laparotomy
54.19	Other laparotomy
54.21	Laparoscopy
54.91	Percutaneous abdominal drainage
54.95	Incision of peritoneum
70.52	Repair of rectocele
70.73	Repair of rectovaginal fistula
86.63	Full-thickness skin graft to other sites
86.69	Other free skin graft
86.7	Pedicle or flap graft, not otherwise specified
86.72	Advancement of pedicle graft
86.74	Attachment of pedicle or flap graft to other sites
86.83	Size reduction plastic operation
96.22	Dilation of rectum
